# User needs gathering for the design of information and communications technology-supported occupational stress management intervention: A quantitative study

**DOI:** 10.1177/20552076221127778

**Published:** 2022-09-21

**Authors:** Manoja Weerasekara, Åsa B Smedberg, Ganga Karunathilaka, Hélène Sandmark

**Affiliations:** 1 Department of Computer and System Sciences (DSV), 7675 Stockholm University, Stockholm, Sweden; 2 Department of Information and Systems Sciences, NSBM Green University, Homagama, Colombo, Sri Lanka; 3 Ramazzini Research Institute, Stockholm, Sweden

**Keywords:** Digital health intervention, occupational stress management, Sri Lanka, software industry, survey, design preference

## Abstract

**Introduction:**

The software industry is considered a growing industry with a high propensity to cause stress reactions among employees due to its demanding and technology-driven nature. There are convincing shreds of evidence that information and communications technology (ICT) interventions can effectively solve stress-related disorders. However, several identified constraints have hindered its potential benefits, such as poor adherence, lack of engagement, high attrition and lack of personalisation.

**Objective:**

The study presented in this article aims to gather the user needs of software employees in Sri Lanka to identify design ideas for an ICT-supported intervention for occupational stress management (ICTSMI) in the software industry.

**Materials and methods:**

The study followed a quantitative approach using an online questionnaire in which three key areas were investigated: (1) stressors, (2) coping strategies and (3) design preferences. A total of 408 valid responses were collected and analysed using SPSS software.

**Results:**

Findings revealed that the majority of software employees were with a moderate level of stress. They perceived work stressors and role stressors as common causes of stress. The most frequently used coping strategy was to seek social support, followed by digital activities and sports and physical exercises. The male and female design preferences varied to a greater extent but only slightly varied based on their job category. However, findings suggested the necessity of further elicitation of user needs to support the design process.

## Introduction

Ensuring healthy lives and promoting well-being at all ages is essential for sustainable development. According to the Sustainable Development Goals (SDG) 2030 agenda, this has been recorded as the third goal: ‘Good Health and Wellbeing’.^1^ Though health comprises physical and psychological facets, the mental health aspect was not covered in the earlier versions of the SDG agendas. However, contemplating the growing need and concerns arising in mental health, in 2015, this was added to the health goal, and this inclusion was considered a historic turning point. The target 3.4 under SDG 2030 health goals requests that: ‘By 2030, reduce by one-third premature mortality from non-communicable diseases through prevention and treatment and promote mental health and wellbeing’.^2^ This inclusion positively impacts communities and countries where millions of people will receive much-needed help to elevate their mental well-being.^3^ Now governments are bound to secure their citizens’ mental well-being, assuring sufficient access to healthcare facilities. However, looking at the current resource availability for mental healthcare, it is always recommended to move with digital platforms and interventions to provide convenience and equal access to mental health resources.^4^

Sri Lanka is a multicultural and multi-ethnic country lying southeast of India, with a 21.94 million population in 2020. The Sri Lankan software industry is considered the fourth largest export revenue segment within the economy.^5^ According to the year 2020 statistics,^5^ the SLASSCOM (Sri Lanka Association for Software Services Companies – the apex body of Sri Lankan software and services companies) marked 600 + information and communications technology (ICT) companies with an 88,051 information technology (IT) workforce. Among them, nearly 60% are involved in software engineering (39%), quality assurance (15%) and business analysis (6%) domains. The industry is mainly male dominant, with 34% of female participation. Out of the total IT workforce, 85% carry a bachelor's degree or above educational qualification. The companies are mainly located in the Colombo area, which is the commercial capital of the country. With the economic targets of Sri Lanka, the SLASSCOM plans to create 200,000 direct jobs, enable 1000 start-ups and grow the IT industry revenue up to USD 5 billion by 2025.^5^ Within the context of a fast-paced industry, stressed employees are inevitable. Thus, both individuals and organisations take numerous measures to combat stress and assure well-being.^6, 7^ Thus, considering the scarcity of sufficient mental health professionals within the country^8^ and rising stress statistics,^9^ digital mental health (DMH) interventions could consider a real game-changer.

Although there is convincing evidence that DMH interventions can effectively solve this problem, several constraints identified in the interventions have hindered its potential benefits. Among them, poor adherence, lack of engagement, high attrition and lack of personalisation are significant.^10^ Moreover, many interventions are developed without focusing on a specific occupational category or any theoretical foundation.^11^ Thus, careful analysis of the user needs and the collaboration of multidisciplinary teams in the design and development process is necessary.^4^ The traditional requirement elicitation techniques used in Software Development Life Cycles (SDLC) are mainly qualitative. These techniques capture and provide only a limited set of qualitative data reflecting individual customer needs.^12^ The captured data might not represent a large user base and does not reveal users’ actual preferences and could lead to the development of interventions not valued by the end user.^13^ World Health Organization's Healthy Workplaces Framework has published generic guidelines on best practices in designing work-related stress management interventions (SMIs).^14^ These guidelines suggest that the content of the intervention should: be based on sound scientific theory; identify psychosocial risks to employee well-being by conducting a risk assessment for work-related stress; tailor the intervention components and tools to the particular occupational sector and the particular organisation; and be implemented systematically with aims, objectives and strategy clearly defined.

**Figure 1.**
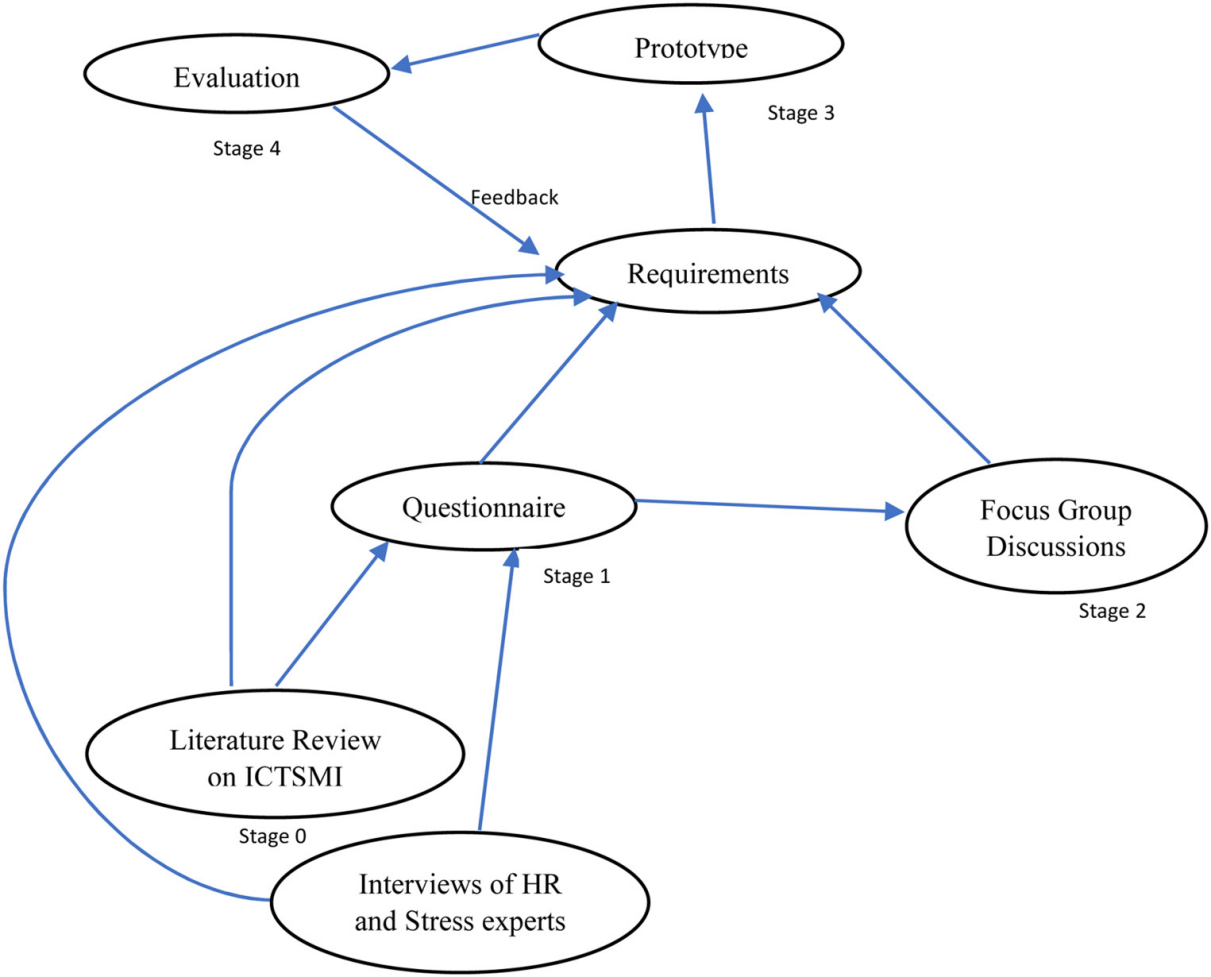
Requirements elicitation process (adopted from^16^).

The study presented in this article could be considered the first stage of the requirements elicitation process to design and develop an ICT-supported intervention for occupational stress management (ICTSMI) in the Sri Lankan software industry. The entire research study is planned to execute in five consecutive stages (see Figure 1).

1. *Stage 0:* The initial stage was carried out in two main activities. First, researchers conducted a literature review to capture the design considerations and research gaps in the existing studies of ICTSMI.^11^ Next, the perception and user experience of human resource (HR) managers^7^ and mental health practitioners^15^ on occupational stress and ICTSMI in Sri Lanka were investigated.
2. *Stage 1:* This is the current study. The aim is to get user needs from a large group of software engineers in Sri Lanka so as to get different views from the prospective users. A questionnaire approach is used.
3. *Stage 2:* In this stage, multiple focus group discussions are planned to further refine the user needs/ design considerations driven from stage 1.
4. *Stage 3:* Based on the input from the earlier stages, the initial prototype will be designed.
5. *Stage 4:* The prototype will be evaluated among the stakeholders (in multiple focus groups) to receive feedback. The feedback will be used to refine the formulated design considerations.

The study presented in this article (stage 1) is quantitative and aimed at capturing a sufficient breadth of the user needs of the study population. However, to gain a deeper understanding of the gathered user needs, findings will be further explored using qualitative focus group discussions in a future study. The selected hybrid approach will provide the opportunity to capture user needs with sufficient depth and breadth over a single requirement elicitation technique.^16^ The proposed hybrid approach reflects several key elements in Yardley's^17^ work on a person-based approach to designing digital health interventions. Stage 0 of the proposed requirements elicitation process reflects the activity set proposed in Yardley's intervention planning stage (examining literature, consultation of experts and synthesising user views on ICTSMI from previous qualitative work). Moreover, the upcoming studies will also reflect the activities proposed in the person-based approach (e.g. creating design principles, mixed-method evaluations, etc.).

The process of the current study (stage 1) is threefold. First, it aims to identify the perceived stress levels and stressors associated with software employees. Next, it investigates the coping strategies and activities practised by the software employees. Finally, it aims to identify the requirements to support the design and development of an ICTSMI. This article is organised as follows. It begins by introducing the areas that frame the study. Then, it describes the research methodology and empirical results. Finally, it concludes by discussing the implications of the research findings for the design of ICTSMI.

## Materials and methods

The study is quantitative and followed a descriptive survey design approach to describe the functional and non-functional requirements (NFRs) of an ICT-supported intervention along with stressors and coping strategies used by software employees. The following sections elaborate on the data collection, the study instruments used, the pilot testing of the study constructs and the study sample and its demographic data.

### Development of the study instrument

The study uses an online survey as the research instrument to capture three objectives: (1) investigate perceived stress levels and stressors, (2) identify coping strategies and activities and (3) gather user needs of the ICTSMI. The questionnaire was developed using the Stockholm University Survey Tool incorporating four main sections. The sections and the sources of constructs are shown in Table 1. Apart from the survey questions section, an introductory page was added to describe the proposed study's scope and aims and then requested the respondent's consent to continue the survey.

**Table 1.** Survey sections and their sources.

Section No.	Content	Source
1	Demographic factors	^18^
2	Stressors and stress level	^19, 20^
3	Coping mechanisms and activities	^19, 21^
4	Perception of ICT-supported intervention and proposed functional and non-functional requirements (NFRs)	^11^

ICT: information and communications technology.

The demographic data, including age, gender, marital status, job category, number of years of working experience, educational qualification, company size and employment status, were obtained in the first section of the survey. The questions in this section were adapted from the General Nordic Questionnaire for Psychological and Social Factors at Work^18^ and modified according to the Sri Lankan setting.

To assess the stressors experienced by the software employees, researchers have declared a model consisting of constructs adopted from the revised version of the WCCL^19^ and Rajeswari and Anantharaman's^20^ work on Indian Software employees’ stress. The proposed explanatory factors are as follows: (1) Work stressors (5 items), (2) role stressors (4 items), (3) personal development stressors (3 items), (4) interpersonal relationship stressors (2 items), (5) organisational climate stressors (3 items) and (6) work–family interface stressors (2 items). All these constructs were formed as 5-point Likert scale questions. The perceived stress level is measured using a 1–10 stress scale where 1 represents no stress and 10 indicates a high-stress level. Later during the analysis, the values were further categorised into three main stress categories mild (1–3), moderate (4–7) and severe (8–10). Along with the perceived stress levels, employees’ sleeping habits and perception of work–family balance are also inquired.

The coping strategies were adopted based on the techniques for managing stress suggested by Michie in his work on Causes and Management of Stress at Work.^21^ Following his work, the researchers identified a list of activities that could be utilised as coping activities. This list contains activities characterised as problem-focused strategies (talk to the supervisor, talk to HR, time management, etc.) and emotional-focus strategies (listening to music, jogging, hanging around with friends and family, using social media, etc.). The Way of Coping Checklist-Revised (WCCL-R)^19^ has five subscales: Problem-focused coping (15 items, e.g. ‘came up with a couple of different solutions to the problem’); Seek Social Support (6 items, e.g. ‘talked to someone about how I was feeling’); Blame Self (3 items, e.g. ‘criticised or lectured myself’); Wishful Thinking (8 items, e.g. ‘hoped a miracle would happen’); and Avoidance (10 items, e.g. ‘went on as if nothing had happened’). Along with the coping activity list, the researchers used the ‘Seek Social Support’ subscale at the WCCL-R to investigate how software employees seek social support when their job becomes a source of conflict and tension, leading to stress.

The final section is the main focus of the study, which leads to the elicitation of the requirements for the proposed ICT intervention. This section contains an author-defined set of questions inspired by the design considerations suggested in a recent literature review^11^ on design practices and implications on ICT-supported SMIs. The survey section starts with preliminary questions investigating respondents’ prior experience and awareness of similar ICTSMI and their willingness to use such interventions for stress management purposes. The following subsections investigate their ‘platform’ preference (e.g. web, mobile and sensors) along with functional requirements. Functional requirements are characterised by two main sub-schemes: activities and support and interaction. These sections provide various options focusing on problem and emotional-focused strategies and support and interaction mechanisms for the user evaluations. The final subsection seeks the user assessment of the NFRs of the proposed intervention.

At the end of the survey and soon after each main section, a text area was provided to receive participants’ ideas and comments that were not covered (online Supplemental material). Through these open answer alternatives, the respondents may formulate their answers in their own words. The strength is that the answers are not limited to the questions and can contribute with a qualitative element.

To ensure the applicability of the survey instruments to the Sri Lankan context, the researchers adjusted questions from earlier studies. These adjustment decisions were mainly based on the insight from the qualitative pilot study and views from the subject matter experts. The pilot study was conducted online using the Zoom platform. The respondents were selected covering all the key job categories, software engineering, quality assurance engineering, business analysis, project management and system support engineering. In the beginning, respondents were invited to fill out the survey. Then, respondents were invited to go through the survey questions from the beginning and provide their feedback and comments. The comments and feedback were recorded both manually and digitally. After the pilot study, researchers amended the survey accordingly based on the insights revealed. Two subject experts examined and validated the survey before the pilot study. Then after the pilot study, the slightly amended version of the survey is re-examined by the same subject experts to assure the validity of the study instrument.

### Survey dissemination and data collection

Permission to conduct the study was sought and granted by the Ethics Review Committee of the Medical Research Institute, Sri Lanka. After receiving the required approval, the survey was launched as a self-administered internet survey using the Stockholm University survey tool. Several channels were utilised to disseminate the survey link. The current study was conducted in Sri Lanka, involving software employees working in software companies located in the Colombo district. The survey link was mainly emailed to the SLASSCOM corporate office, requesting to share the link with its member organisations. Then the link was sent to HR managers of eight software companies (SLASSCOM listed) who participated in an earlier study. The HR managers were requested to share the link with their employees. The survey link was also disseminated among five Facebook groups of Sri Lankan Software employees. Since Facebook is the most popular social media platform among Sri Lankans, the selection of this channel enabled us to reach a larger audience conveniently. The survey was available for 2 months (30 October 2020 to 31 December 2020) and collected 489 responses. Out of the 489 responses, 81 partially completed responses were excluded from the analysis.

### Data analysis

Data were analysed using the IBM SPSS Statistics Version 27. Given the larger sample of responses received, the statistical assumption of the normality of the data was examined using the normal probability plots. The survey‘s internal consistency is measured using Cronbach's alpha, and it was valued at 0.77. The data were presented as frequencies and percentages for categorical data and mean and SD for continuous data. Significant differences in the stress levels with the sociodemographic and occupational characteristics were tested using an independent *t*-test and one-way between-groups Analysis of variance (ANOVA) with post hoc tests for categorical data. The analysis mainly focused on identifying the main stressors, coping strategies and design requirements for the proposed intervention.

## Results

### Characteristics of the subjects

A total of 408 complete responses were returned on or before the deadline of the data collection period. Among the subjects, 89 (21.8%) were female, and 206 (50.5%) were single. More than 60% of the respondents were involved in software engineering, quality assurance and UI/UX engineering positions. The majority of the employees belonged to the age group of 25–34 years (n = 298, 73%). Their job experience ranged from 0 to 22 years, with a mean of 6.3 years (SD = 3.7). Most of them (n = 297, 72.8%) were bachelor's degree holders. Among the subjects, 87% (n = 354) were permanent employees, and 61% (n = 247) were employed in large-scale organisations. The demographic characteristics of the subjects are summarised in Table 2.

**Table 2.** Demographic profile of the respondents.

Variables	Frequency (%)
Gender	
Male	319 (78.2)
Female	89 (21.8)
Age (years)	
18–24	34 (8.3)
25–34	298 (73.0)
35 and above	76 (18.6)
Marital status	
Single	206 (50.5)
Married	194 (47.5)
Single (divorced or widowed)	8 (2.0)
Education qualification	
Up to higher diploma level	20 (4.9)
Bachelor	297 (72.8)
Postgraduate	91 (22.3)
Job category	
Software engineer (SE)	159 (39.0)
Quality assurance engineer (QA)	68 (16.7)
User experience engineer (UI/ UX)	71 (17.4)
Project manager (PM)	33 (8.1)
Business mnalyst (BA)	24 (5.9)
Implementation and support engineer (SUP)	22 (5.4)
Database administrator (DBA)	31 (7.6)
Years of work experience (years)	
0–3	101 (24.8)
4–8	193 (47.4)
9 and above	113 (27.8)
Employment type	
Contract	54 (13.2)
Permanent	354 (86.8)
Size of the company	
Small (1–49 employees)	78 (19.1)
Medium (50–99 employees)	83 (20.3)
Large (100 employees and above)	247 (60.5)

**Figure 2.**
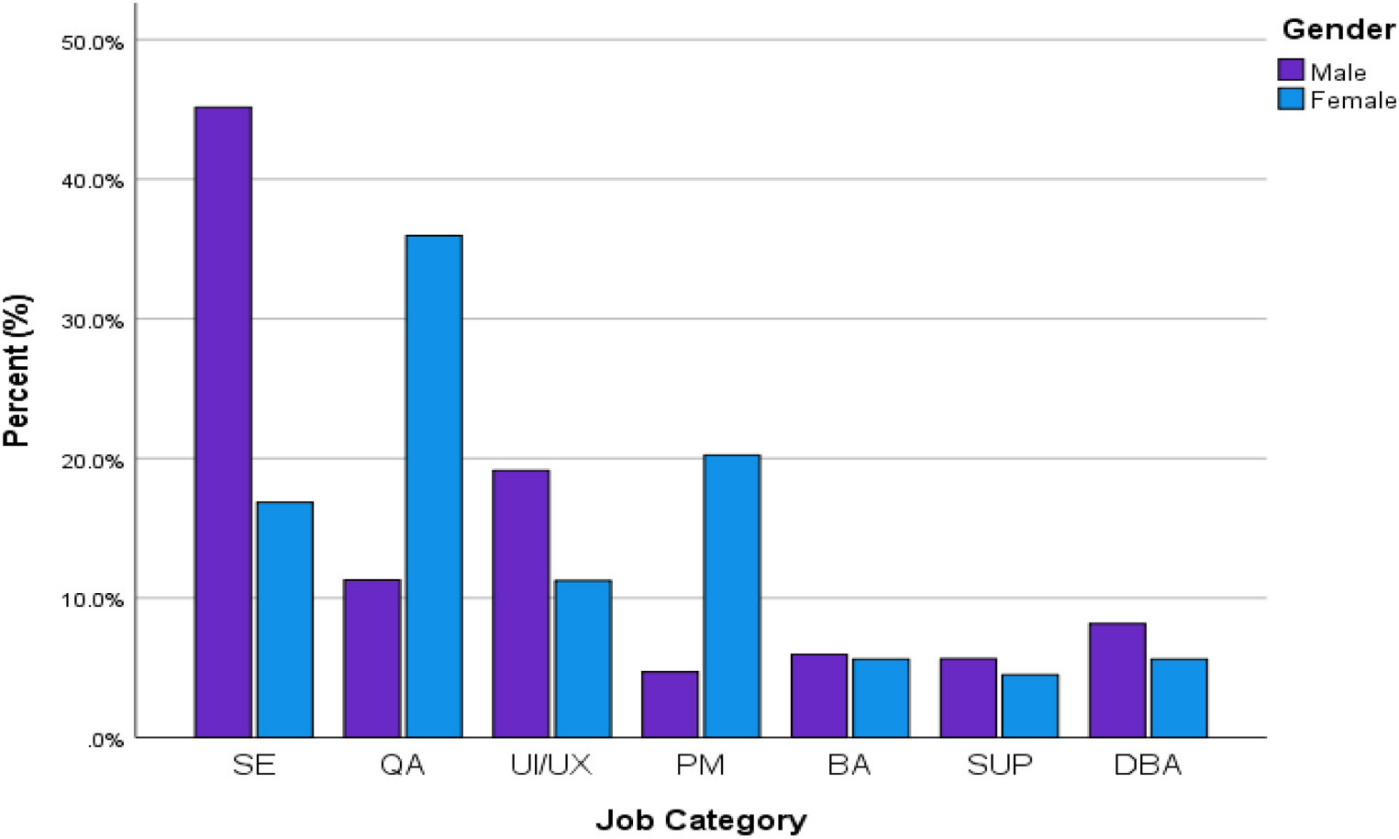
Job category and gender wise distribution of the respondents.

The following chart (Figure 2) shows the job category and gender wise distribution of the respondents. There were more male software engineers than females, but female representation is higher in both QA and PM disciplines.

### Stressors and stress levels

In this study, the perceived stress level ranged from 1 to 10 with a mean of 6.37 (SD = 1.78), suggesting that the software employees experienced a moderate frequency of job stress. Most of them (n = 265, 65.0%) fall into the moderate stress level (in the range of 4–7), and 26.7% (n = 109) of employees indicated severe stress levels (in the range of 8–10). The mean scores of each stress factor ranged from 3.43 to 3.90, indicating that software employees perceived (agreed) all the factors as contributors to their stress levels. They perceived work stressors (M = 3.97) as the highest source of stress, whereas personal development stressors (M = 3.49) and work–family interface (M = 3.43) were rated as the least sources among them (refer to Table 3).

**Table 3.** Mean scores of the stress categories.

Stress category	Mean	SD
Work stressors	3.97	0.68
Role stressors	3.73	0.84
Organisational stressors	3.61	1.01
Personal development stressors	3.49	0.93
Work–family stressors	3.43	0.96

An independent samples *t*-test was used to compare the mean score on stress levels among males and females. Test results showed no statistically significant difference (*p* = 0.669) in male and female mean stress level scores. Moreover, multiple iterations of one-way between-groups ANOVA with post hoc tests were carried out to test whether there are significant differences in the mean scores on stress levels across sociodemographics. Table 4 shows the statistical output on mean score differences of perceived stress level between the groups of age, job category, years of experience, education level, years of experience and size of the software company. There was a statistically significant difference in stress levels between different job categories as determined by one-way ANOVA (*F* (6401) = 2.271, *p* = .036). A Tukey post hoc test revealed a statistically significant difference in stress levels between quality assurance engineers and UI/UX engineers. The test indicated that the mean score for UI/UX engineers (M = 6.87, SD = 1.51 95% CI = 6.52–7.23) was significantly different from quality assurance engineers (M = 5.90, SD = 1.51 95% CI = 5.53–6.26). However, the resulting eta squared value is .03, which Cohen's^22^ (pp. 284–287) terms would be considered a small effect size. There was no significant statistical difference identified between the other job categories. One-way ANOVA revealed a marginally significant main effect of years of experience and perceived stress levels, *F* (2404) = 2.895, *p* = .0.56.

**Table 4.** Result of ANOVA for statistically significant differences in stress level among groups.

Grouping condition	df	Mean square	F-value	Sig
Age	2	2.431	0.760	0.468
Job category	6	7.117	2.271	0.036*
Educational level	2	2.910	0.911	0.403
Years of experience	2	9.134	2.895	0.056
Size of the company	2	0.128	0.040	0.961

### Coping strategies

There are seven main coping strategies identified, representing both problems focused and emotion-focused strategies. The mean scores of using each coping strategy are listed in Table 5. The most frequently used coping strategy was to seek social support, followed by digital activities and sports and physical exercises. The analysis of the individual activities revealed a high mean score (M = 3.70) for social media usage. Mental and physical relaxation and substance abuse were rated as the least coping mechanisms.

**Table 5.** Mean scores of the coping strategies.

Coping strategy	Mean	SD
Seek social support	3.31	1.08
Digital activities	3.12	0.96
Sports and physical exercises	2.79	0.92
Self-distraction activities	2.57	0.69
Religious activities	2.01	0.91
Mental and physical relaxation activities	1.85	0.83
Substance abuse	1.95	1.25

There was a statistically significant difference in coping mechanisms among job categories as determined by one-way ANOVA. Test results revealed a statistically significant difference in self-distraction (*F* (6401) = 2.851, *p* = .010) as the coping mechanism among software engineers and project managers. Post hoc comparisons using the Tukey HSD test indicated that the mean score for software engineers (M = 2.44, SD = 0.66, 95% CI = 2.34–2.55) was significantly different from project managers (M = 2.84, SD = 0.78, 95% CI = 2.56–3.12). Similarly, there was a statistically significant difference between quality assurance engineers and business analysts using substances (*F* (6401) = 2.693, *p* = .014) as the preferred coping mechanism. The Tukey HSD test indicated that the mean score for quality assurance engineers (M = 1.67, SD = 1.13, 95% CI = 1.40–1.95) was significantly different from business analysts (M = 2.16, SD = 1.37, 95% CI = 1.58–2.74). The software engineers, UI/UX engineers and project managers identified a statistically significant difference using religious activities (*F* (6401) = 5.467, *p* = .000) as the preferred coping mechanism. The mean comparisons of these three groups indicated a high mean score for the project managers (M = 2.66, SD = 1.08) than UI/UX engineers (M = 1.91, SD = 1.00) and software engineers (M = 1.84, SD = 0.89). Though the Tukey post hoc test revealed statistically significant differences in the coping mechanisms used by different job categories, such differences were with a small effect size.

Further, an independent samples *t*-test is used to compare the mean score on each coping strategy among males and females. Test results showed a statistically significant difference in males and females using sports and physical exercises (*p* = 0.011) and substance abuse (*p* = 0.000) as their preferred coping mechanisms. According to the results, males preferred engaging in physical exercises and substance abuse over females.

Table 6 shows the means scores retrieved from the sources where employees seek support when the job becomes stressful. Accordingly, employees preferred solving the problems by themselves (M = 4.08), followed by seeking support from co-workers (M = 3.17) and seeking support from supervisors (M = 2.91). The least scored source of support is seeking support through HR (M = 1.89). Software employees preferred engaging in stress management activities individually (M = 3.71) over in group (M = 2.51).

**Table 6.** Mean scores of the support-seeking mechanisms.

Seek support from	Mean	SD
Myself	4.08	1.01
Co-workers	3.17	1.07
Supervisors	2.91	1.00
Family and friends	2.64	1.23
Human resource division (HR)	1.89	1.00

### Requirement gathering for the design of intervention

The following section elaborates on the functional and NFRs suggested by the respondents regarding the ICT-supported stress management intervention. It is worth noting that only 24.5% of the respondents had experience with similar applications, and only 40.4% are aware of digital interventions for stress management purposes. However, 64.5% of respondents were interested in using ICT-supported interventions, whereas 10% of them indicated their unwillingness and 25.5% doubted their decision.

#### Preference for intervention platform

The analysis of preferences on the platform revealed mobile (M = 3.66) as the preferred platform to render the SMI, followed by the hybrid (web and mobile) approach (M = 3.05) and web platform (M = 2.96). Table 7 shows the mean scores received on each platform.

**Table 7.** Mean scores of the design preferences.

Preference on	Item	Mean	SD
Platform	Mobile application	3.66	0.737
	Web application	2.96	0.960
	Sensor or wearable device-based application	2.70	1.123
	Desktop application	2.33	1.121
	Hybrid application	3.05	1.001
Features	Audio and video module	1.52	0.771
	Gaming module	1.83	0.974
	Motivational feedback module	1.72	0.892
	Physical exercise module	1.91	0.887
	Mental relaxation activity Module	1.58	0.774
	Text-based conversation module	2.29	1.049
	Voice-based conversation module	2.17	0.996
	Video-based conversation module	2.16	1.073
	Chatbot	2.33	1.104
	Social networking platform within the organization	2.10	1.032
	Social networking platform outside the organization	2.01	0.979
Support from	counsellor	3.84	1.173
	Peers	3.96	0.922
	Automated	3.51	1.175
	Self-help	4.12	0.977
NFRs	Confidentiality of the activities and data	4.63	0.745
	Security mechanisms	4.57	0.806
	Content accuracy	4.58	0.744
	Tailor-made content facility	4.30	0.824
	Interactivity	4.31	0.888

#### Preference for features

Respondents marked on a 4-point Likert scale indicating their preferences on the stress management activities proposed in the intervention. The mean scores ranged from 1.52 to 2.33, indicating their preferences in the vocal range of very little to somewhat. Table 7 shows the mean scores received on each feature.

#### Preference for support & guidance

Upon the provided support mechanisms, the self-help option (M = 4.12) was rated as the most preferred mode, followed by support from peers (M = 3.96) and support from counsellors (M = 3.84). The automated support option received a mean score of 3.51. Table 7 indicates the mean scores and SDs received on each mode.

#### Preference for NFRs

Respondents highly valued the non-functional features of the proposed intervention. The mean scores ranged from 4.12 to 4.63, indicating substantial agreement on the proposed NFRs. Respondents highly valued the confidentiality of the actions and data (M = 4.63), followed by the content accuracy (M = 4.58) and security (M = 4.57). Table 7 shows the means scores and SDs on each feature.

Moreover, the analysis of open-ended questions on design preferences revealed the respondents’ preference for screening and assessing stress levels, monitoring and tracking activities and predictive contents.

#### Design preferences by gender, age and job category

Along with the overall analysis of the respondents’ design preferences, the effect of gender and age and job category was analysed using suitable statistical methods.

The effect of gender was analysed using an independent samples *t*-test to compare the mean scores on design among males and females. Test results showed a statistically significant difference in some platform preferences, feature preferences, support preferences and preferences on NFRs in males’ and females’ mean stress level scores. Males preferred physical exercises, conversations and gaming components over females. The female group's mean scores were high in support-seeking mechanisms, whereas females preferred to seek support from peers and counsellors over their counter group. It is essential to highlight that female has higher mean scores for NFRs than males. Table 8 shows the means scores extracted for the statistically significant differences in design preferences by gender.

**Table 8.** Statistically significant design preferences based on gender.

Design preferences		Male	Female
Platform	Mobile	3.61	3.84
	Desktop	2.27	2.54
	Hybrid	2.98	3.27
Features	Audio video	1.57	1.35
	Gaming	1.90	1.58
	Motivational feedback	1.81	1.42
	Physical exercises	1.98	1.65
	Mental relaxation activity module	1.66	1.27
	Text-based conversation module	2.40	1.87
	Voice-based conversation module	2.25	1.88
	Chatbot	2.45	1.88
	Social networking platform within the organization	2.17	1.85
Support From	Counsellor	3.75	4.15
	Peers	3.90	4.16
	Automated	3.43	3.81
NFRs	Confidentiality of the activities and data	4.58	4.78
	security Mechanisms	4.50	4.81
	Content accuracy	4.53	4.74
	Tailor-made content Facility	4.26	4.47
	interactivity	4.26	4.48

The effect of age on design preferences was analysed using ANOVA. The test results showed no statistically significant differences in the mean scores on design preferences across the three age categories. However, the job category effect on design preferences analysis revealed significant differences between groups (Table 9). Though the Tukey post hoc test revealed statistically significant differences in the coping mechanisms used by different job categories, such differences were with a small effect size. Implementation engineers preferred (M = 2.91) desktop-based intervention over UI/UX engineers (M = 2.03). Considering the feature preferences, the software employees preferred (M = 2.02) gaming features more than the business analyst (M = 1.38) and both software engineers and UI/UX engineers preferred the voice-based conversation feature more than the project managers (M = 1.73). The support-seeking behaviour indicated that both QA engineers (M = 4.15) and project managers (M = 4.27) preferred peer support over the software engineers (M = 3.75). However, there was no significant difference identified in the preference for NFRs among the groups.

**Table 9.** Result of ANOVA for statistically significant differences in design preferences among job categories.

Design preference		df	Mean square	F-value	Sig.
Platform	Desktop	6	3.757	3.08	0.006
Features	Gaming	6	2.197	2.363	0.030
	Motivational Feedback	6	2.486	3.223	0.004
	Voice Conversation	6	3.196	3.331	0.003
Support	From Peers	6	2.199	2.647	0.016

#### Insights from open alternatives

An inspection of the responses provided at the open alternatives was carried out to identify further suggestions that the analysis might not cover. Participants have not provided any comments on the main sections but have provided some answers to the last open-ended question. Out of the 408 responses, 60 replies carried such comments and feedback. A minor content analysis of the data revealed several user requirements that can be categorised into three main categories (Table 10). The first category received most of the responses in which the respondents sought the possibility of embedding more features they wanted to see in the proposed intervention and the possibility of extending the collaboration with peers. The second category covered the users’ emphasis on adding technologies like artificial intelligence and machine learning into the intervention. They were also interested in making the intervention more automated by capturing measures from sensors. Finally, the third category highlighted user emphasises on selecting the right visual elements and maintaining simplicity.

**Table 10.** Findings from the analysis of open alternative.

Category (user requirements)	Sample quotes extracted from the responses
More features and collaboration with peers	‘Analyze patterns, historical data of activities/stress levels, and set/maintain goals.’ ‘A function to quantify how much I am stressed and what for then recommending remedies’ ‘A platform where people can share their stories about how they managed to handle similar situations and overcame stress in different situations.’ ‘Share activities on social media platforms.’ ‘Allow users to upload content videos. eg- playing piano pieces or share paintings with others and interact with them’
Embedding emerging technologies (e.g. AI, sensors and data mining)	‘Predictive content (like mood rings, but instead to see what kind of factors stress them out)’ ‘Tracking both mental and physical health by using key indicators such as sensors’
The simplicity of the user interfaces and processes	‘Just want a simple solution’ ‘Should easy to use - User Experience, UI also need to be catchy’ ‘A very calm and serene UI’

## Discussion

The study focused on gathering requirements for the design and development of ICTSMI. The researchers gathered data from 408 respondents covering all the scales of software companies and job categories available in the Sri Lankan context. Information collected is analysed to get insightful information on the key requirements.

The demographic data of this study revealed that most of the software employees work as software engineers attached to large-scale companies. The gender disparity is also observed where a majority of the respondents were male. A relatively young employee population was identified who are with bachelor's level qualifications. This distribution aligns with the recent statistics published by the SLASSCOM for 2021 on the IT industry in Sri Lanka. According to the report,^5^ 66% represent males in the IT workforce. Looking at the composition of the IT workforce by service lines, 39% work as software engineers and 15% work as quality assurance engineers. Out of the 600 + companies listed in SLASSCOM, most of the workforce belongs to large-scale software companies.^5^ Thus, it concludes that the sample represents the current context of the Sri Lankan software industry.

Continuing the recent growth in the IT segment and Sri Lankan government targets, the IT-BPM industry is well poised to reach an export revenue of USD 3 billion by 2025.^5^ Within this context, software employees are in challenging work settings in terms of (1) global pressures: market and rivalry, (2) technology pressures: rapid tech-advancements and (3) local pressures: working hours and changing work culture due to its global software development nature.^23^ However, the results revealed that the job stress level of the IT professionals was generally moderate, with some experiencing a high level of stress. The study findings also indicate that work stressors and role stressors were the most frequent stressors experienced by Sri Lankan Software employees. These were consistent with the previous results reported in similar work settings involving IT professionals.^20, 24^

Analysis of coping strategies used by the software employees revealed that the most frequent coping strategy was seeking social support followed by digital activities and physical exercises. Extending the analysis, study results on support-seeking sources indicated that software employees frequently relied on themselves or sought support from their co-workers. A similar phenomenon is identified in the results of the support preferences of the DMH interventions as well. The respondents preferred to follow the self-help mode followed by support from co-workers and counsellors. The observed self-reliant nature of software employees is also reflected in the study of Rajeswari and Anantharaman^20^ involving software employees in India. With the unequal representation of gender, the IT industry seems to be one of the most male-dominated sectors. Thus, traditional masculine characteristics such as independence, self-reliance and dominance^25^ may also be impacted by the high preference for the self-reliant nature of the software employee. Computer self-efficacy and intrinsic motivation may have built the self-belief to take risks and deal with professional obsolescence and may also have contributed to the same.^20^ Even though individualism is showcased, the software development process functions using teamwork towards a given target. These individuals’ performances affect the entire team‘s performance. Diversities in individual competencies might deteriorate a team's performance. In such a situation, individuals have to take responsibility to learn and handle the situation or sometimes need to seek support from their co-workers.^20^ It implies that more competent team members have to take on multiple roles, which causes role overload.^20^ In addition, tight time schedules and a shortage of skilled personnel increase the team members’ workload.^20^

Moreover, it is interesting to highlight the gender impact on support-seeking behaviour. The results showed a significant difference in the peer support preference among quality assurance engineers and project managers over the software engineers. The quality assurance engineers and project managers were more interested in seeking peer support than the software employees. The cross-analysis highlighted that in both QA and PM categories, there were more female respondents. Thus, a high percentage of female respondents may have impacted this behaviour. Several studies on the impact of gender on support-seeking behaviour reveal the same. They have highlighted that a lower rate of help seeking in men is often attributed to traditional masculinity stereotypes, including self-reliance and restrictive emotionality.^26, 27^

The study findings revealed that the least preferred source of support seeking was the HR of the company. A study conducted in Sri Lanka involving HR managers in software companies also reported this gap identified between the software employees and the HR.^7^ The same study reported that only a minority of employees report grievances or problems to HR, and most of them reveal their problems at the offboarding. The respondents’ high preference for social media usage is worthwhile discussing. High social media usage in society might have ignited social media usage in work settings and for work purposes. This phenomenon is shared across all work settings. A recent study^28^ conducted on social media usage by employees and companies showed two main types of social media platforms: corporate/enterprise social media (e.g. Microsoft Yammer) and personal social media (e.g. Facebook, WhatsApp and WeChat). However, the current study revealed a high usage of personal social media, characterised as socialisation-oriented social media. Such platforms enable social and personal information exchange and facilitate expressive ties that influence individual identity through social and emotional support and normative expectations.^28^

Study findings indicated that mobile is the most preferred platform, followed by the hybrid (mobile and web) platform. This preference aligns with the current Sri Lankan and global statistics on mobile penetration and internet usage.^29^ In Sri Lanka, 64% of the web traffic is generated by mobile devices, and a share of 98.7% of the social media users is accessing them via a mobile platform.^29^ Thus, the availability of the resources, familiarity and ease of use might have influenced the selection of the platform preference. As discussed in the questionnaire development phase, the feature list suggested in the survey was extracted from studies of DMH interventions and existing applications for stress management. However, respondents show only mean scores ranging from 1.52 to 2.33, indicating their preferences in the vocal range of very little to somewhat. This implies their feature preference is different from what is proposed or available in the current DMH interventions. Thus, in-depth discussion with the respondents is suggested to understand their needs fully. If the user need is not adequately understood, it will lead to design and development interventions that the end users do not appreciate.^13^ A recent review on the DMH intervention also highlighted the problem of untailored and unresponsive features.^13^ Such limitations had caused high attrition, low adherence, low fidelity and disengagement with the intervention.^11, 13^ It is interesting to note the high mean scores received on the proposed NFRs set over the proposed functional requirement. This highlights the importance and user preference given to such feature content. Respondents highlight their need for embedding confidentiality, security, reliability, usability and performance into the DMH interventions. Several studies on DMH interventions also highlighted the importance of embedding NFRs.^30, 31^ Moreover, a comprehensive study^32^ on multi-stakeholder design considerations on mobile-based health interventions also indicates the importance of addressing NFRs. However, the same study indicated how such requirements could limit the available platform preferences and implementation choices while increasing the development time.

The study's preliminary findings shed light on the development of the following design considerations (Table 11). These six design considerations will be further explored during the subsequent study.

**Table 11.** Preliminary design considerations.

Derived design considerations [Focus on ….]	Description
Modality and intervention level	The intervention delivery platform and the target level (individual, group, hybrid, organisational, etc.) need to be selected at the initial stage.
Content Provision	The intervention needs to carefully select the techniques and technologies to deliver the right content to the user in an efficient way.
Personalisation	The system needs to render personalised content based on the user's requirements, acknowledging their familiarity and experience.
Social Connectedness	The intervention needs to enable the user to connect with social circles of their choice (peers, experts, etc.)
Confidentiality and security	Needs to pay attention to assure the confidentiality of the user data associated with the application. The collected data should be securely stored prohibiting unauthorised access.
Aesthetics and Simplicity	The intervention needs select visual elements to offer pleasing aesthetics while assuring the simplicity of the intervention.

In the current context of software development, feature content in the system tends to be prioritised using limited volumes of qualitative user input.^13^ This may lead to the development of feature sets undesirable for the end users as the target audience consists of a heterogeneous group of individuals. In the current study, intended end users constitute a broad group of software employees in different occupations and companies in the IT industry. Thus, gathering user needs from a small sample will not provide a detailed depiction of the user needs. On the other hand, using a quantitative approach enables a larger volume of requirements to be collected to support the design and development.^8^ Thus, the current study utilises a quantitative method to understand the user needs and requirements in the pre-development stage. This is useful for identifying design ideas for a suitable prototype of an ICT intervention. A qualitative approach using multiple focus group discussions is planned during the next stage to refine further the design ideas gathered from this study. Such discussions allow researchers to gather more insights from the participants.^33^ The combination of survey and focus group discussions makes it possible to use the strengths of quantitative and qualitative approaches to define the pre-development stage requirements.^13, 16^ Since the study highlighted a significant difference in design preferences perceived by males and females, focus groups will be formed to capture this difference. Then, based on the requirements gathered and insights from literature and similar applications, the prototype of the ICT intervention is to be developed.

This study has some limitations, which would threaten the potential benefits and validity of the study. The main limitation observed is the approach taken to design the questionnaire. As elaborated in the survey design section, the researchers developed questions to capture user needs and preferences based on the findings from the previous studies. None of the requirements elicitation questions for the proposed ICT intervention has been extracted or adopted from previously published questionnaires. This could threaten the quality of the data gathered since the questions have not been extensively tested before. However, the pilot testing and the expert survey review have supported to assure that the questions are accurate measures of the concept of interest The resulting Cronbach's alpha value also guaranteed the internal consistency of the survey. The quantitative approach to exploring user needs could also be a limitation. However, since open answer alternatives were also added to the questionnaire, the respondents could formulate some of the answers themselves, in their own words; however, more focus on the analysis of open answers could give further strength to the data collection and contribute to a qualitative element.

The data was collected on one occasion, so the causality is unclear. The direction of causality is somewhat clearer regarding background data on the number of children, age at birth of the first child, number of employers and earlier jobs, but more unclear regarding reports of other factors in working life. As the study is based on retrospective self-reported data, the reporting could be dependent on experienced stress-related problems and a decreased health status. This is a limitation due to the design of this study, and some of the reported aspects of working life could have been influenced by stress among the respondents. But this was what they reported at the time of our investigation, and it reflects the circumstances when reporting. The factors investigated could hardly be estimated in any other way than by self-report, and therefore, the conclusions should be drawn with care.

Though the data was captured from a larger audience, the findings’ depth or details are limited. Thus, multiple focus group discussions are planned in the subsequent study to explore the findings further. The study involved a more extensive sample that represents the composition of the gender, age and occupational category of the target population; the study findings can be generalised to the population. Thus, it assures the external validity of the study. Since the Sri Lankan software industry resembles the global software industry's work culture, the findings may also generalise to a broader geographical area.

## Conclusions

This article investigated user needs for the design and development of ICT-supported occupational SMIs. The survey study revealed different stressors and coping strategies used by software employees. These findings could provide insights for employers to rethink the possible SMIs and what protocols to embed in their work settings. The design preferences identified in the study signal the importance of understanding the users beyond their demographics. It highlighted the importance of involving more qualitative studies as proposed in Yardley's^17^ work on the person-based approach to capture the individual preferences and keep the whole end-to-end user experience of the proposed intervention. To overcome the drawbacks and limitations of the single requirement elicitation technique, in future work, the gathered user needs could be further refined through in-depth focus group discussions. The outcome of this study may offer insights for IT professionals working in the design and implementation of ICT-supported SMIs and contribute to the DMH knowledge domain and researchers involved in designing ICT-based mental health interventions.

## Supplemental Material

sj-docx-1-dhj-10.1177_20552076221127778 - Supplemental material for User needs gathering for the design of information and communications technology-supported occupational stress management intervention: A quantitative study Click here for additional data file. Supplemental material, sj-docx-1-dhj-10.1177_20552076221127778 for User needs gathering for the design of information and communications technology-supported occupational stress management intervention: A quantitative study by Manoja Weerasekara, Åsa B Smedberg, Ganga Karunathilaka and Hélène Sandmark in Digital Health
