# Supplementary material for: User needs gathering for the design of information and communications technology-supported occupational stress management intervention: A quantitative study
Source: Digit Health. 2022 Sep 21;8:20552076221127778. doi: 10.1177/20552076221127778 (PMC9500269; doi:10.1177/20552076221127778)
Supplement: sj-docx-1-dhj-10.1177_20552076221127778 - Supplemental material for User needs gathering for the design of information and communications technology-supported occupational stress management intervention: A quantitative study [file sj-docx-1-dhj-10.1177_20552076221127778.docx]

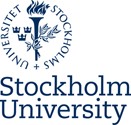


The purpose of this study is to investigate current stress management practices use by software employees and to understand their perception of ICT supported occupational stress management interventions. The study only focus on the stress associated in the working envioronment.

**As an employee of the software industry** we kindly request your honest information, and thoughtful suggestions to make this objective a sucess.

This survey will take only 5-8min to complete.

We are very appreciative of the time you take to assist in our study. Thank You So Much...!!

1. **I'm a**


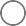


Male Female


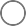


1. **I belong to age category of**


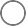


18-24 Years

25-34 Years

35- 44 Years

45-54 Years

55-64 Years

65 and over


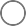

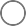

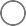

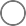

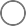


1. **What is your marital status?**


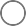


Married

Single, Never Married Single, Divorced Single, Widowed


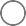

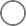

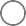


**4. If you have children living at home, please specify the no. in respective age categories.**

No Children

Less than 4 years old 4 to 12 years old

13 to 18 years old

19 and over

1. **Highest Education Qualification that you have**


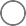

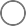

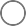


1. **My job title?**


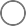


GCE A/L or Less

Certificate/Diploma/ Higher Diploma level Bachelor’s Degree

Postgraduate qualification (Postgraduate Diploma, Masters, PhD,etc.)

Other

**7. I have….. years of experience in the software industry**

1. **My employment is on**


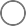


Contract Basis Permanent Basis


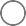


1. **Size of the company**


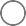


Small (Less than 49 employees) Medium (50-99 employees)

Large (More than 100 employees)


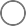

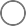


1. **Do you think that your family-work life is well balanced?**


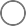


Yes

To a certain extent No

Not sure


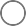

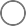

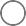


1. **What is your habitual sleep like?**


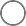


Very Calm Fairly Calm

Neither Calm nor Restless Quite Restless

Very Restless


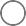

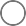

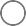

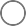


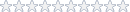


**12. How would you rate your average stress level at work 1-No Stress and 10- Highest**

1. **What makes you stressed at your work (Select Where Applicable)**


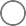

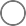

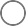

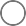

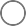

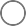

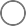

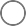

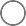

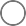

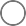

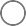

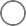

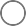

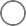

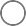

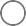

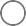

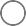

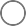

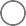

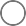

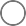

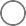

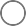

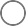

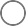

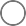

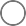

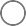

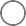

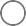

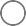

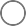

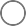

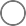

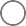

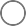

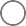


Strongly Agree Agree Neutral Disagree Strongly

Disagree


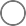

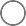
Long Working Hours
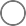

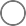
 Unmanageable Workload
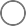

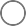
 Tight deadlines and Poor

Deadline Management


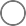

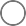
Poor Relationships with Colleagues


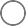

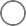
Rapidly Changing Nature of Technology & Requirement (

Stay up-to date with technology)


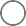

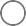
Unsupportive Boss or Organizational Culture


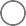

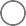
Few Career Growth Opportunities


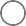

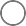
Assuming Different Roles in the Same Project or in Different Projects (Disturbances)


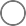

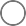
Lack of Technical Expertise
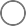

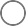
 Lack of Support from Family

or Family Commitments

Presure being a team

manager/leader and/or
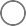

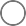
 managing subordinates


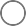

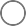
Gray Areas on development requirements


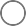

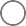
Client Interaction/ Pressure Arise from the Clients (from both internal and external

parties)

Strongly Agree Agree Neutral Disagree Strongly


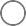

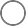

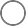


Disagree

Poor Project Management

Insufficient Monetary/ Non- monetary Incentives/

Transport and Other Benefits

Organizational Setting and Work Environment

Insifficient Leave Facility (Casual, Medical, Annual, Parental Levales, etc)

Problem of handling multiple responsibilities (In work as well as in the family)

Other

1. **In case of stressful situation what are the activities you involve in to reduce your stress levels?**

Rarely (Never) Occasionally Sometimes Fairly Often Very Often

(Always)

Listen to music Watch videos or movies Engage in religious activities Practice Yoga Do meditation Walk Practice Deep breathing Practice Muscle relaxation

activities

Do Singing and/or Dancing Do Painting Hanging around with friends

and family (Go on trips, partying, chit-chat, etc.)

Play digital games (computer

games, online games, mobile games, etc.)

Use social media (Use FB, WhatsUp, Tweeter,etc)

Engage in Physical Activities/Sports (e.g. Badminton, Cricket, Swimming)

Smoking/Consume Alcohol Other

Rarely (Never)

Occasionally

Sometimes

Fairly Often

Very Often (Always)

Talk to my supervisor/boss and request support

Talk to HR and request support

Talk to co-workers and request support

Talk to friends and family members and request for support

Try to solve it by your own

**15. When your job becomes a source of conflict and tension leading to stress, you will**

Rarely (Never)

Occasionally

Sometimes

Fairly Often

Very Often (Always)

Individually/Alone

With a close friend or Partner In a group

As an online activity

As an offline (Physical Activities)

Comment

**16. How do you usually perform activities to reduce your stress levels? (select all applicable)**

**17. Do you know about any ICT tools available for stress management? (E.g. Websites/mobile apps/Online forums/etc.)**

Yes No

**18. Have you ever used such applications?**

Yes No

**19. Are you interested in using ICT supported tools to manage your stress levels?**

Yes No

Not Sure

1. **If you are interested to use ICT supported tools to manage your stress level, what is your preferred platform?**

To a Great Extent Somewhat Very Little Not at all

Mobile applications Web application Wearable sensors

Standalone application (Desktop applications)

Hybrid (combination of above options)

1. **What features would you like to see on such platform? (select all applicable)**

To a Great Extent Somewhat Very Little Not at all

Audio and Video clips Gaming components

Motivational and feedback notifications

Physical Exercises

Mental relaxation activities Text or voice-based

conversations Chat Bot

Online Communication Platform/ Social Networking Platform to connect with

people **inside** your organization

Online Communication Platform/ Social Networking Platform to connect with

people **outside** your organization

Other Please specify:

Very Important

Important

Moderately Important

Slightly Important

Not Important

Confidentiality Security

Content Accuracy

Customizable/tailor made content

Interactive components

Other,Please specify

**23. What are the other features that you find important in ICT applications for stress management?**

**22. What type of online support that you prefer in ICT supported tool to manage stress [Support and interaction in the form of: Messages, feedback, conversations, guidance, etc.,]**

Very Important

Important

Moderately Important

Slightly Important

Not Important

Support from a counsellor

Support from

Peers/Colleagues/Friends Computer based Guidance

Self-help or manage by myself option

**24. Please specify other features and functions in ICT supported tools that might help you to manage high stress level & motivate you to use such system**
